# Electroacupuncture Preconditioning Improves Myocardial Infarction Injury via Enhancing AMPK-Dependent Autophagy in Rats

**DOI:** 10.1155/2018/1238175

**Published:** 2018-08-06

**Authors:** Qing Zeng, He He, Xian-Bao Wang, Yu-Qing Zhou, Hong-Xin Lin, Zhi-Peng Tan, Shang-Fei He, Guo-Zhi Huang

**Affiliations:** ^1^Department of Rehabilitation Medicine, Zhujiang Hospital, Southern Medical University, 253 Industrial Avenue, Guangzhou 510282, China; ^2^Department of Cardiology Medicine, Zhujiang Hospital, Southern Medical University, 253 Industrial Avenue, Guangzhou 510282, China

## Abstract

**Background:**

Electroacupuncture (EA) pretreatment plays a protective role in myocardial infarction injury. However, the mechanism of electroacupuncture remains unknown. The aim of this study was to confirm the protective effects of electroacupuncture (EA) on myocardial infarction injury and the possible mechanism.

**Methods:**

Sprague-Dawley (SD) rats, used to serve as acute myocardial infarction (AMI) model, were divided into sham group, model (M) group, M+EA group, AMPK inhibitor Compound C (M+EA+CC), and AMPK inhibitor solvent control (M+EA+DMSO) group, respectively. Rats in EA group were pretreated with EA and those in M+EA+CC group with intravenous AMPK inhibitor Compound C. The myocardial morphological changes and infarct size were observed through HE staining and TTC staining, and the concentrations of CK-MB and LDH were detected using ELISA kits. Transmission electron microscopy was employed to observe the autophagosome formation, and the AMPK-dependent autophagy-related protein expression was detected by immunohistochemistry and western blot.

**Results:**

EA could alleviate myocardial infarction injury and decrease the concentrations of CK-MB and LDH. Transmission electron microscopy showed that EA could also regulate the AMPK-dependent autophagosome formation and the AMPK-dependent autophagy-related protein expression. AMPK inhibitor Compound C could impair the effect of EA through regulating the concentrations of CK-MB and LDH, autophagosome formation, and autophagy-related protein expression.

**Conclusion:**

These results indicated that electroacupuncture could improve myocardial infarction injury and induce autophagy, and AMPK-dependent autophagy might be involved in this process.

## 1. Introduction

Myocardial infarction resulting from coronary arterial occlusion leads to myocardial necrosis, contractile dysfunction, arrhythmias, and even heart failure; ischemic heart disease is the major cause of mortality worldwide [1]. Although numerous traditional treatments and drugs have been used, damaged myocardial cells remain nonrenewable and scarred myocardial tissue still cannot be restored completely [2]. Therefore, many researchers are studying more effective alternative therapies. Kataoka et al. found that omentin could prevent myocardial infarction injury [3], and the vestigial enzyme D-dopachrome tautomerase could protect the heart against ischemic injury [4]. Some studies demonstrate that stem cell therapy could prevent cardiomyocytes apoptosis, promote local neoangiogenesis, improve myocardial perfusion, and reduce the local inflammatory response in the acute phase of MI [2, 5]. And transplantation of stem cells following AMI has been tested in clinical trials [6].

Acupuncture, one of the most well-known complementary and alternative medical approaches, has a long history of clinical practice and has been applied to prevent and cure diseases, including depressive disorder [7], stroke [8], and ischemic heart diseases [9]. Previous studies indicated that acupuncture at Neiguan (PC6) acupoint exerted cardioprotective effects on ischemic heart injuries, including AMI [10], myocardial ischemia-reperfusion, and cardiac surgery [11]. And, acupuncture preconditioning at Neiguan (PC6) acupoint, as a form of acupuncture, has a protective effect on myocardial ischemia-reperfusion injury in rabbits and rats [12–14]. The mechanisms of the above researches focused on biochemistry and the neuroendocrine system (e.g., energy metabolism, oxidative stress, mitochondrial function damage, myocardial cell apoptosis, and microcirculation disorders) [15].

Autophagy is a process that degrades cytoplasmic constituents and has diverse physiological functions, including intracellular protein degradation, starvation, adaptation and organelle clearance, immunity, and cell death; it is a bulk degradation process in eukaryotic cells and plays a fundamental role in cellular homeostasis [16]. Autophagy is commonly observed in the heart with acute and chronic ischemia and heart failure [17]. In the repetitive myocardial stunning model, autophagy was observed in the myocardium with less apoptosis, and heart function was fully recovered after normalization of the coronary flow, which suggested that autophagy might promote survival of myocardium [18]. 5′-AMP-activated protein kinase (AMPK) is a critical regulator of autophagy in vivo during myocardial ischemia, and activation of AMPK is always accompanied by stimulation of autophagy in cardiac myocytes [19].

Based on the previous study, acupuncture preconditioning at Neiguan (PC6) acupoint verifiably protects against myocardial infarction injury [12–14], yet no studies have focused on autophagy. Therefore, in this study, we prepared the AMI model in vivo to confirm the protective effects of EA on AMI injury, and whether EA regulates autophagy in AMI injury.

## 2. Materials and Methods

### 2.1. Animals

Sixty adult, male Sprague-Dawley (SD) rats (200-220 g) were utilized in this experiment. The rats were obtained from the Experimental Animal Center of Southern Medical University, Guangzhou, China. Both the animal care and the study protocol were conducted according to the Guide for the Care and Use of Laboratory Animals by the National Institutes of Health, in addition to the guidelines of the Ethics Committee of Southern Medical University. All surgeries were performed under chloral hydrate anesthesia, with efforts to minimize suffering. The rats were housed under a 12-hour light/dark cycle at a constant room temperature of 24 ± 1°C.

### 2.2. Treatment and Preparation of AMI Model

Adult male SD rats were randomized into the following groups: sham group, model group (M group, without any other treatment), EA group (EA preconditioning + model group), AMPK inhibitor group (M+EA+CC, EA preconditioning + model group + intravenous AMPK inhibitor Compound C), and AMPK inhibitor solvent control group (M+EA+DMSO, EA preconditioning + model group + intravenous administration of an equal volume of DMSO), with 6 rats in each group. The acupoints aimed for acupuncture were Neiguan (PC6) acupoint located inside the forelimb, and 15 × 0.32 mm needles ware stabbed subcutaneously 2-3 mm into the two acupoints simultaneously using SDZ-II type electroacupuncture treatment instrument (output voltage: 2-4 V, output current: 4-6 mA), 30 min each time, once a day, with a total period of 7-day continuous EA before AMI modeling. Before each EA treatment, AMPK inhibitor group rats were given intraperitoneal injection AMPK inhibitor Compound C (dissolved in DMSO and the original concentration of Compound C was 250 g/kg; dilution with normal saline when used, dosage is 100 *μ*g/kg/day, a total of 7 days). And, AMPK inhibitor solvent control group rats were given intraperitoneal injection DMSO in the same manner as above.

Then, all the rats were anesthetized using 10% chloral hydrate (400 mg/kg) and intubated, before a left thoracotomy was performed in the fourth intercostal space; AMI was then induced by permanent ligation of the left anterior descending coronary artery with a 6/0 braided silk suture [20]. A standard II-lead electrocardiogram (ECG) was performed for monitoring cardiac changes, and an artificial ventilator was used to assist breathing during the experiments. When the ligation of the above-mentioned parts of the heart became white, the pulses weakened and ECG ST segment arched significantly upward, which meant the modeling was successful. After modeling success, the researchers immediately collected the blood and took the heart. The rats, which had only performed the thoracotomy, but not been treated with permanent ligation of the left anterior descending coronary artery, served as sham group control.

### 2.3. TTC Staining

The rats were narcotized by intraperitoneal injection of 10% chloral hydrate (400 mg/kg), before the hearts were removed and stained using 2,3,5-triphenyltetrazolium (TTC) staining. Briefly, the hearts were isolated and cut into 4-mm slices, which were immediately immersed in 1% TTC solution (Sigma Chemical Co.) in phosphate buffer (pH 7.4) at 37°C for 15 min. After being washed three times, the slices were photographed, and the infarct area was compared with the total area by use of digital planimetry software (Image-Pro Plus 6.0).

### 2.4. Hematoxylin and Eosin (HE) Staining

Each heart was fixed in 4% paraformaldehyde-phosphate buffered saline (PBS) for 30 min and cut into 5 *μ*m sections. Slides were then stained following the following steps: 70% ethyl alcohol (EtOH) for 10 s; diethylpyrocarbonate-treated water for 5 s; hematoxylin with RNAase inhibitor for 20 s; 70% EtOH for 30 s; eosin Y in 100% EtOH for 20 s; and xylenes for 2 min (after dehydration with a series of alcohol for 30 s each). Finally, after being washed three times, the histological structure of myocardial tissues was observed with microscope (400×).

### 2.5. Transmission Electron Microscopy Assay

The hearts were perfused with 4% paraformaldehyde and double-fixed in 1.5% glutaraldehyde and 1% osmium tetroxide. After being dehydrated in acetone, the hearts were treated in propylene oxide and embedded in EMbed 812/Araldite. Ultra-thin slices were prepared using LEICAUC6 slicer, and then double-stained with uranyl acetate and lead citrate. Finally, a Philips electron microscope (Philips, Amsterdam, Dutch) was used to observe the ultrastructure of myocardium and the formation of autophagy.

### 2.6. Quantitative Detection of CK-MB and LDH

Before taking the heart, the researchers collected the blood of the heart and separated the serum after centrifugation. The concentrations of creatine kinase MB isoenzyme (CK-MB) and lactate dehydrogenase (LDH) were measured using ELISA kits. The absorbance (OD) values of all samples were measured at 450 nm wavelength, and the concentrations of CK-MB and LDH were calculated through the standard curves.

### 2.7. Immunohistochemistry Assay

The cardiac muscle tissues were fixed in 10% buffered formalin and cut into 5 *μ*m sections, which were deparaffinized and subjected to heat-induced epitope retrieval. Slides were then incubated with primary antibodies at 37°C for 2 h. The antibodies were detected using the avidin-biotin-peroxidase complex method, and the sections were counterstained with hematoxylin. The staining intensity was measured for each sample. Blank group served as the negative control. Mean optical density (MOD) was calculated using Image-Pro Plus 6.0 software (Media Cybernetics, USA).

### 2.8. Western Blot Assay

After brief sonication, the cardiac muscle tissues were centrifuged at 12,000 rpm for protein collection. The supernatants were collected, and the protein concentration was determined using a BCA protein assay kit. For western blotting, altogether 10 *μ*g protein was subjected to a 12% sodium dodecyl sulfate-polyacrylamide gel electrophoresis (SDS-PAGE) and subsequently transferred to PVDF membrane (Millipore, Bedford, MA, USA). The membranes were blocked and then incubated with target primary antibodies (LC3B: Abcam, ab48394, dilution 1:200; Becline-1: Abcam, ab207612, dilution 1:2000; Bcl-2: Abcam, ab196495, dilution 1:500; P62: Abcam, ab91526, dilution 1:50; p-AMPK: Abcam, ab109402, dilution 1:1000; AMPK: Abcam, ab131512, dilution 1:500; p-mTOR: Abcam, ab137133, dilution 1:1000; mTOR: Abcam, ab2732, dilution 1:2000) at 37°C for 2 h. After washing, the membranes were incubated with horseradish peroxidase-labeled goat anti-rat secondary antibody (Abcam, ab6721, dilution 1:2000) at 37°C for 1 h. Finally, the signals were detected after incubation with a chromogenic substrate using the enhanced chemiluminescence (ECL) method. Additionally, GAPDH served as the internal control. The mean gray values of each protein band were measured by Photoshop CS5 software (Adobe, San Jose, CA, USA). The relative band intensity is the ratio of the gray value of the protein of interest to that of the corresponding GAPDH.

### 2.9. Statistical Methods

SPSS17.0 statistical software was utilized for statistical analysis. The data were presented as the mean ± standard deviation (SD). The significance of the differences was determined by Student's* t*-test or one-way analysis of variance (ANOVA) with the least significant difference post hoc test when equal variances were assumed or with Dunnett's T3 post hoc test when equal variances were not assumed.* P*<0.05 was considered statistically significant in comparison.

## 3. Results

### 3.1. EA Prevented Histological Structure Changes of Myocardial Tissues and Reduced Myocardial Infarction Size

**Figure 1:**
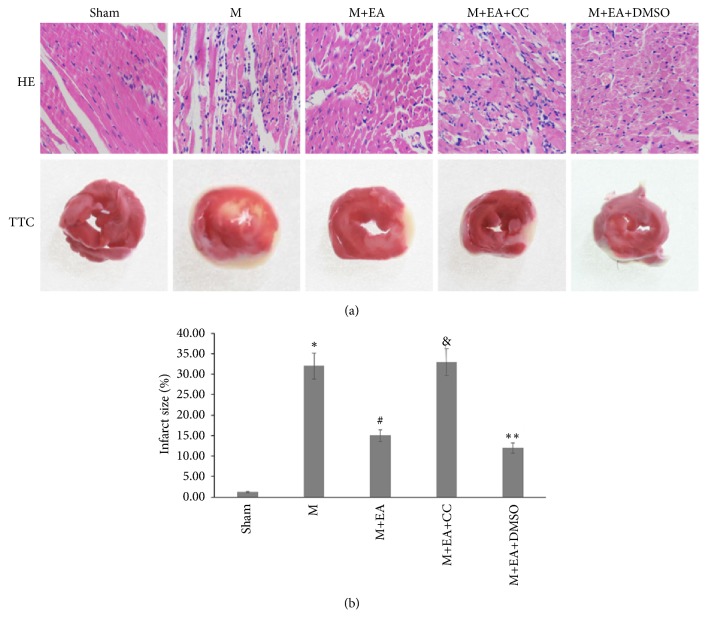
Observation of cardiomyocyte morphology and infarct size. (a) HE staining and TTC staining of myocardial tissues in each group (400×). (b) Statistical analysis of infarct size in each group. The data are presented as mean ± SD (n = 6). Sham group versus M group, ^*∗*^*P*<0.05; M+EA group versus M group, ^#^*P*<0.05; M+EA+CC group versus M+EA group, ^&^*P*<0.05; M+EA+DMSO group versus M+EA group, ^*∗∗*^*P*>0.05. Scale bar: 50 *μ*m.

HE staining (Figure 1(a)) showed that the myocardial cells in the sham group remained well-arranged and had normal nuclear structure. However, in the model group the myocardial cells were loosely connected and some were in disarray, with interstitial inflammatory exudation being significantly increased. After EA treatment, the myocardial cells were arranged more neatly and interstitial inflammatory exudation decreased. On the contrary, AMPK inhibitor Compound C rendered EA treatment ineffective.

After staining with TTC, the heart tissue in sham group was red and part of the myocardium ischemic tissue turned gray. Moreover, the myocardial infarct size decreased after EA treatment as the red staining area enlarged significantly (Figure 1(b),* P*<0.05). Inversely, AMPK inhibitor Compound C led to enlargement of the infarct size and ineffectiveness of EA treatment. The results indicated that the AMI model was successfully established; EA treatment could alleviate myocardial infarction; and AMPK pathway was involved in the regulatory process of EA.

### 3.2. EA Reduced the CK-MB and LDH Expression

As shown in Table 1, in comparison with sham group, the concentration of CK-MB and LDH in model group was significantly increased (*P*<0.05). And after EA preconditioning, the concentration of CK-MB and LDH decreased significantly (*P*<0.05). In addition, when AMPK pathway was blocked by AMPK inhibitor Compound C, the concentration of CK-MB and LDH increased to the level close to the model group (Table 1). These results demonstrated that EA could regulate the concentrations of CK-MB and LDH, and AMPK pathway was involved in this process.

### 3.3. EA Induced the AMPK-Dependent Autophagosome Formation

**Figure 2:**
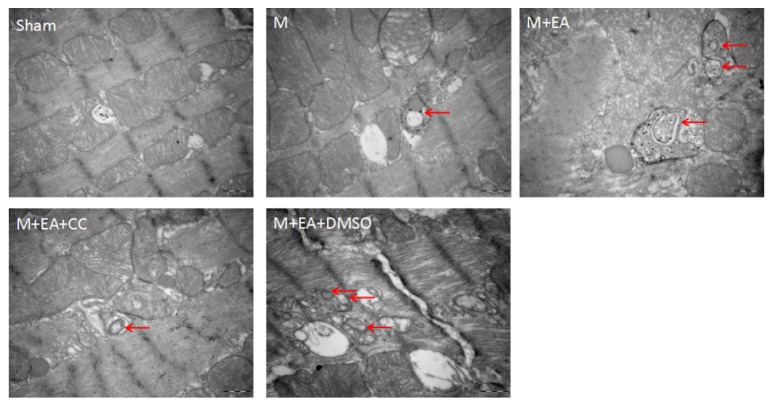
Observation of autophagosomes by electron microscopy in each group. The red arrows point at autophagosomes. Scale bar: 0.5 *μ*m.

An autophagosome is a bilayer or occasionally multiple membrane structure which contains undegraded cytoplasm. As illustrated in Figure 2, no autophagosome was found in the sham group, and the number of autophagosomes increased significantly in the model group. After EA treatment, the number of autophagosomes rose significantly, and a typical bilayer membrane structure and undegraded cytoplasm were found. AMPK inhibitor Compound C treatment reduced the number of autophagosomes. The result of transmission electron microscopy images indicated that EA could regulate the AMPK-dependent autophagosomes formation.

### 3.4. EA Increased the AMPK-Dependent Autophagy-Related Proteins Expression

**Figure 3:**
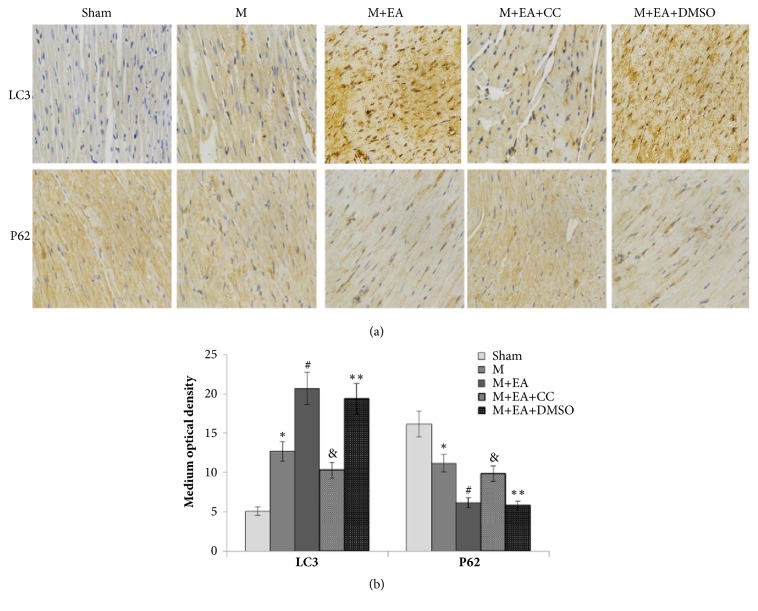
Immunohistochemistry analyses of autophagy-related proteins. (a) Immunohistochemistry images of LC3II and P62 in each group. (b) Statistical analysis of medium optical intensity of each group. The data are presented as mean ± SD (n = 5). Sham group versus M group, ^*∗*^*P*<0.05; M+EA group versus M group, ^#^*P*<0.05; M+EA+CC group versus M+EA group, ^&^*P*<0.05; M+EA+DMSO group versus M+EA group, ^*∗∗*^*P*>0.05. Scale bar: 50 *μ*m.

Immunohistochemical staining showed that LC3 was expressed in the cytoplasm (Figure 3(a)). Compared with sham group, the LC3 medium optical intensity in model group was significantly increased from 5.1 ± 0.6 to 12.7 ± 1.1 (Figure 3(b),* P*<0.05). After EA treatment, the LC3 expression increased (*P*<0.05) and AMPK inhibitor Compound C could lead to ineffectiveness of the EA treatment. In addition, EA treatment could downregulate the expression of P62, and AMPK inhibitor Compound C could weaken this downregulation.

**Figure 4:**
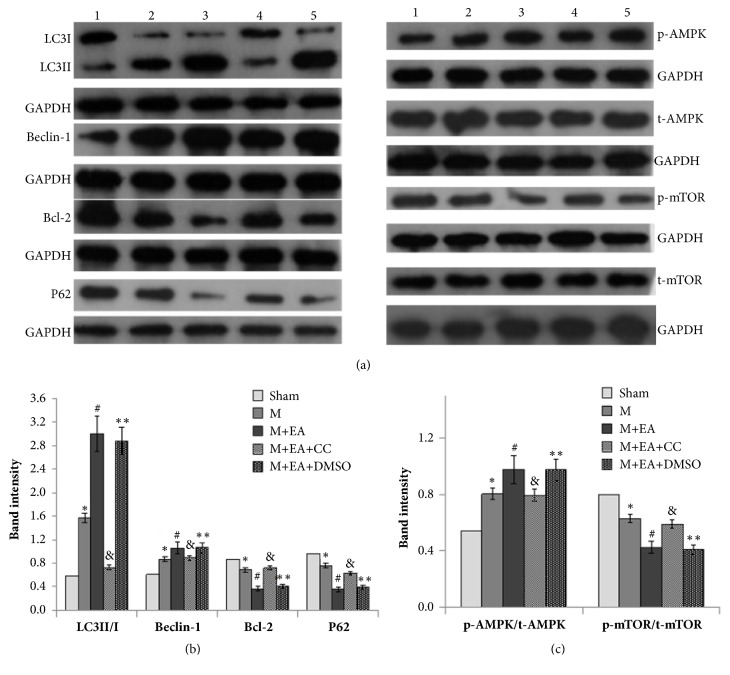
Western blot analyses of autophagy-related proteins. (a) Schematic image of western blot in each group. (b) Gray scale analyses of the relative LC3II/I, Beclin-1, Bcl-2, and P62 expression levels. (c) Gray scale analyses of the relative p-AMPK/t-AMPK and p-mTOR/t-mTOR expression levels. The data are presented as mean ± SD (n = 6). Sham group versus model group, ^*∗*^*P*<0.05; EA group versus model group, ^#^*P*<0.05; M+EA+CC group versus M+EA group, ^&^*P*<0.05; M+EA+DMSO group versus M+EA group, ^*∗∗*^*P*>0.05.

LC3II/I ratio and Beclin-1 expression level were increased in the model group in contrast to sham group, and EA treatment could significantly increase the LC3II/I ratio and Beclin-1 expression level (Figures 4(a) and 4(b),* P*<0.05). On the contrary, EA could decrease the Bcl-2 and P62 expression. Moreover, western blot result showed that the expression phosphorylated p-AMPK was upregulated in model group compared with sham group, and EA treatment induced upregulation of p-AMPK expression. Additionally, phosphorylated p-mTOR expression was downregulated in the model group as opposed to sham group, and EA treatment could reduce the p-mTOR expression. Total t-AMPK and t-mTOR expression level remained unchanged among five groups. On the whole, AMPK inhibitor Compound C could impair the effect of EA on the above-mentioned protein expression (Figures 4(a) and 4(c)). These results revealed that EA could regulate the AMPK-dependent autophagy-related protein expression.

## 4. Discussion

Acupuncture is being increasingly accepted as an alternative medical therapy worldwide due to its verifiable effectiveness. According to traditional Chinese medicine, the acupoint Neiguan is located above the wrist, covering one-sixth of the distance from the distal wrist crease to the cubital crease, between the palmaris longus tendon and the flexor carpi radialis tendon. In this study, Neiguan is defined to be located in the forelimbs based on the textbook of experimental acupuncture in animals [21]. Neiguan is not only the luo-connecting point of pericardium meridian relating to Sanjiao meridian, but also one of the eight confluence points interlinking with Yinwei meridian. The fact that Neiguan connects to three meridians contributes to its high value of clinical application and experimental study. A number of clinical studies have shown that acupuncture at Neiguan may alleviate MI symptoms as it can decrease heart rate, lower blood pressure, reduce oxygen consumption, increase coronary blood flow, and improve hemorrheology [22]. It is also found in animal experiments that electroacupuncture at Neiguan can regulate the median nerve, the spinal dorsal horn neurons of C3-T3, and the nucleus of the solitary tract in the brain stem. Electroacupuncture at Neiguan may also adjust vasoactive substances, such as thromboxane B2, prostacyclin I2, endothelin, and calcitonin gene-related peptide [23]. Therefore, we chose the PC6 acupoint for our further research, and based the treatment parameter (sparse-dense wave, output voltage: 2-4 V, output current: 4-6 mA, 30 min each time, once a day) on previous studies [10].

TTC staining has been extensively applied to stain myocardial tissue obtained from humans and experimental animals, and it accurately reflects the extent of irreversible myocardial infarction damage [24]. HE staining, as a traditional histopathologic staining method, can precisely and reliably distinguish infarcted from normal tissue and assist in observation of myocardium tissue morphological changes [25]. Lactate Dehydrogenase (LDH) and MB fraction of creatine kinase (CK-MB) analyses have conventionally been used in humans to assess ischemia-induced cardiac injury. Elevated expression of CK-MB and LDH showed a statistically significant positive correlation in clinical practice [26]. In our AMI model rats, TTC staining demonstrated that the infarct size significantly decreased in the EA group, while it increased in the AMPK inhibitor Compound C group. In addition, HE staining indicated that EA treatment could improve the myocardium tissue morphological damage induced by AMI modeling. Moreover, the concentrations of CK-MB and LDH significantly increased in the model group, and EA treatment could reduce CK-MB and LDH contents. Hence, we conclude that EA can improve myocardial infarction injury in model rats.

The death of a cell is associated with autophagy, which is normally observed in the heart with acute or chronic ischemia and myocardial infarction injury [27]. Repetitive myocardial stunning model indicated that autophagy could be observed in the myocardium with less apoptosis, and heart function was fully recovered after normalization of the coronary flow, which also suggested that autophagy might promote survival of myocardium [15]. Autophagy is controlled by autophagy-related genes (Atgs), which are largely involved in autophagosome formation. Beclin1 (Atg6), as a mammalian autophagy gene, is an essential participant in autophagy. Beclin1 and Bcl-2 family function as a point of cross-talk between the autophagic and apoptotic pathways, and cardiac Bcl-2 transgenic expression also inhibits Beclin1-dependent autophagy in murine heart cells [28]. The conversion of the soluble form of LC3 (LC3-I) to the autophagic vesicle-associated form (LC3-II) is utilized as a marker of autophagy [29]. Using GFP-LC3 transgenic mice, some researchers found that the number of autophagosomes and LC3II/I ratio were significantly increased by ischemia alone and were further increased after I/R treatment [30]. Bjørkøy et al. pointed out that inhibition of autophagy led to an increase in p62 protein levels, as well as autophagic degradation of the p62 via direct interaction with LC3 [31]. In our AMI model, the LC3II/I ratio and Beclin-1 levels increased, while Bcl-2 and P62 levels decreased in the EA group, which manifested that EA treatment could induce autophagy. Combined with the result showed above, we conclude that EA treatment could improve myocardial infarction injury and promote survival of myocardium by increasing the number of autophagosomes.

Previous studies have suggested that mTOR is inhibited during energy starvation and that inhibition of mTOR stimulates autophagy, and inhibition of mTOR is sufficient to induce autophagy [32]. Glucose deprivation resulted in a significant increase in the AMPK phosphorylation, coincident with decreases in the phosphorylation of mTOR, so AMPK-mTOR pathway is thought to be an important regulator of autophagy in response to starvation [33]. In the transgenic mice with cardiac specific expression of DN-AMPK (Tg-DN-AMPK) model, AMPK is activated during ischemia [16]. In our study, the phosphorylation level of AMPK increased, which coincided with mTOR phosphorylation decrease in the AMI model rats. After AMPK inhibitor Compound C treatment of the AMI model rats, the AMPK phosphorylation decreased while mTOR phosphorylation increased. In addition, AMPK inhibitor Compound C could impair the effect of EA on the autophagy-related proteins, AMPK phosphorylation, and mTOR phosphorylation. It is illustrated that apoptosis in the myocardium was inhibited by electroacupuncture mediated autophagy through the increased phosphorylation of AMPK and decreased phosphorylation of mTOR. Therefore, we infer that AMPK-mTOR signaling pathway is involved in the regulation of electroacupuncture mediated autophagy, which is positively correlated with apoptosis in myocardial infarction injury.

In conclusion, this study found that electroacupuncture at PC6 played a part in AMPK-dependent autophagy process and might prevent myocardial infarction injury and may help to provide a rational target for treatment of myocardial infarction injury. Further research is needed to study the other autophagy-related pathways, in order to confirm the effect of electroacupuncture on myocardial infarction/reperfusion injury and find out whether electroacupuncture regulates autophagy process in myocardial I/R injury.

## Figures and Tables

**Table 1:** The concentrations of CK-MB and LDH in each group (n = 6).

	Sham	M	M+EA	M+EA+CC	M+EA+DMSO
LDH (IU/L)	393.17±24.83	1845.50±74.31*∗*	1241.17±49.58*∗*^#^	1768.45±68.96*∗*	1175.62±36.45*∗*^#^
CK-MB (U/L)	66.00±24.04	97.50±29.97*∗*	71.33±22.58*∗*^#^	94.67±20.25*∗*	72.34±25.69*∗*^#^

Compared with sham group, ^*∗*^*P*<0.05. Compared to model group, ^#^*P*<0.05.

## Data Availability

The data used to support the findings of this study are available from the corresponding author upon request.
